# The Effect of an 8-Week Rope Skipping Intervention on Standing Long Jump Performance

**DOI:** 10.3390/ijerph19148472

**Published:** 2022-07-11

**Authors:** Chao-Fu Chen, Hui-Ju Wu

**Affiliations:** Physical Education College, Huaibei Normal University, Huaibei 235000, China; chenchaofu@chnu.edu.cn

**Keywords:** sports performance, exercise, kinematics, biomechanics, strength and conditioning

## Abstract

The purpose of this study was to explore the utility of an 8-week rope skipping intervention in enhancing standing long jump performance was assessed by means of specific kinematic parameters acquired by 3-D space photography. The fifteen male college students from the physical education institute were randomly recruited as the research subjects. Participants first completed a standing long jump test without rope skipping intervention. Participants subsequently took part in a second standing long jump test after rope skipping training. Two high-speed digital cameras with 100 Hz sampling rate were synchronized to capture the movement. The captured images were processed using motion analysis suite, and the markers attached to joints on images were optical auto capture. Based on the results, the velocity of the center of gravity at take-off and landing were significantly improved. In addition, the study confirmed the requirement for forward tilt of the hip joint at landing to increase the velocity of the center of gravity and hence long jump distance. The detailed kinematic analysis described here provided further evidence of the benefits of integrating non-specialized and specialized training activities to enhance athletic performance and offers a contribution to movement theory and practice.

## 1. Introduction

Standing long jump is an important indicator in physical fitness. The best performance is obtained when athletes coordinate the strength in their lower limbs and hip muscles in conjunction with a swinging motion of their upper limbs [1]. Research has revealed an interrelationship between joint angle, angle of the center of gravity, and velocity of the center of gravity at take-off and during the flight phase [2]. Consequently, coordination of movement and technical skills greatly impacts the final performance [3].

Standing long jump is one of the physical fitness test in junior and senior high school and university in many countries. In China, it is one of the test items for the entrance examination for junior and senior high school, and it is also one of the graduation thresholds for every university student [1]. Therefore, the standing long jump performance is very important, and it is also an important factor in measuring physical fitness. Besides, rope skipping exercises the whole body that can improve body mass index, cardiorespiratory endurance, motor coordination, agility, speed, force and balance ability, and plays a prominent role in the improvement of athletic ability [4,5,6,7,8,9]. The activity is extremely safe, requires little space, is inexpensive, and can be performed at home. Moreover, rope skipping offers clear advantages for a wide range of participants including health-conscious adults, exercising teenagers, specialist athletes, and growing children [4,10,11,12,13].

At present, rope skipping is predominantly used as a research tool in studies of physiological response and rarely in corrective kinematic analysis. In contrast, the standing long jump is often used to detect and evaluate the physical fitness of schoolchildren, teenagers, adults, and specialist athletes through human movement analysis [14,15]. As stated above, the purpose of this study was the utility of an 8-week rope skipping intervention in enhancing standing long jump performance of teenagers was assessed by means of specific kinematic parameters acquired by 3-D space photography.

## 2. Materials and Methods

### 2.1. Participants

The fifteen male college students from the physical education institute were randomly recruited as the research participants (height 178.13 ± 3.94 cm; weight 74.33 ± 16.00 kg; age 19.07 ± 0.70 years). Before the experiment commenced, the participants were informed of the purpose of this study and were required to sign a letter of consent. The study complied with the Helsinki Declaration, and the study was approved by Huaibei Normal University, NO: [2021] 78.

### 2.2. Equipments and Data Collection

In this experiment, participants first completed a standing long jump test (T1) without rope skipping intervention. Participants subsequently took part in a second standing long jump test (T2) after rope skipping training. Training was conducted for 8 weeks, 3 times per week and for 30 min per session (Table 1). During these 8 weeks, except for the addition of rope skipping training, all other activities of daily living were maintained. Movement intensity was stipulated according to American College of Sports Medicine suggestions, each participant was required to reach moderate intensity (65–75% of Maximum heart rate) and would start the next round after their heart rate had decreased to 120 beats/minute at the rest interval [16]. Heart rate was measured using the Xiaomi band (Xiaom, XMSH11HM, Beijing, China). Maximum heart rate with reference to Gellish’s equation = 206.9 − 0.67 × age [17,18,19].

Intervention T1 and T2 standing long jump motion analysis, and used two high-speed cameras (sampling rate = 100 Hz, shutter speed = 1/1000 s, Sony, PXW-FS7H, Tokyo, Japan) set at the center position of the three-dimensional coordinate frame respectively at a 45-degree angle and extending 12 m to the side, an LED light acts as a camera synchronization signal. A three-dimensional coordinate frame 2.5 × 2.0 × 2.5 m^3^ (Length × Width × Height) (Peak Motus) in size and including 12 markers was set up with its origin at the center of the taking the long jump take-off. The optical axis of the lens was directed to the center of the coordinate system and the shooting range covered the coordinate system. A total of 21 optical capture reflective markers were adhered to the participant’s head, right and left ear, middle fingertips, joints of shoulder, elbow, wrist, hip, knee, ankle, heels, and toe after the participants had warmed up for 10 min. Each participant performed three standing long jumps at maximum effort, followed by a10-min rest break. The best performance was selected for analysis.

### 2.3. Data Processing

The captured images were processed using Kwon3D motion analysis suite (Visol, Inc., Gwangmyeong-si, Kyonggi-do, Korea), and the markers attached to joints on images were optical auto capture. The X, Y, and Z axes of the global coordinate system represented the horizontal left–right, forward–backward, and vertical upward–downward directions of the space, respectively. Adolescent human limb segment parameters were established with reference to past literature [20].

**Figure 1:**
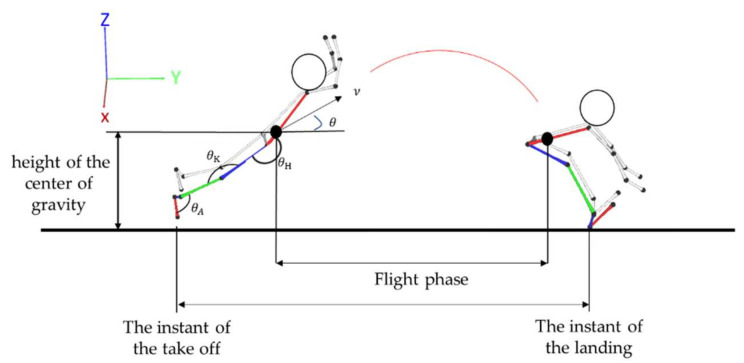
Schematic diagram of the center of gravity and the joint angle. Note: *ν*: velocity of the center of gravity; *θ*: angle of the center of gravity; *θ*_H_: hip joint angles; *θ*_K_: knee joint angles; *θ_A_*: ankle joint angles.

Definition of various kinematic parameters: the velocity of the center of gravity (the resultant velocity $\vec{Rv}$ of the horizontal velocity ${\vec{V}}_{x}$, the forward–backward velocity ${\vec{V}}_{y}$, and the vertical upward–downward directions velocity ${\vec{V}}_{z}$, the angle of the center of gravity between ${\vec{V}}_{x}$, and the resultant velocity $\vec{Rv}$, the height of the center of gravity (the vertical height in the sagittal plane). Hip joint angle (the angle of the lines connecting the shoulder to the hip joint and the hip to the knee joint), knee joint angle (the angle of the lines connecting the hip to the knee joint and the knee to the ankle joint), ankle joint angle (the angle of the lines connecting the knee to the ankle joint and the ankle to the toe (Figure 1). The data were filtered by applying the 4th butterworth low pass filter with the cut-off frequency at 6 Hz before all kinematic parameters were calculated.

**Figure 2:**
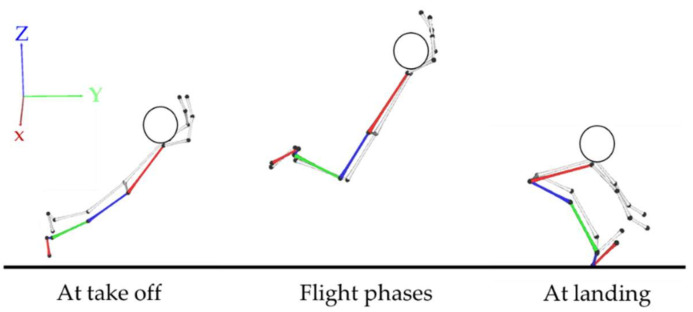
Definition of the phases.

In this study, standing long jump was divided into three phases: (1) At take off: the instant the feet leaves the ground (toes). (2) Flight phases: the instant the feet leaves the ground to the feet landing the ground. (3) At landing: the instant the feet landing the ground (heel). (Figure 2) [21,22].

### 2.4. Statistical Analysis

SPSS 17 was used for data analysis. Using basic descriptive statistics (means, and standard deviations) for all kinematic parameters, then Wilcoxon Signed Rank Test was used for testing the 8-week skipping training intervention to correct the difference in kinematic parameters between T1 and T2 in the three phases of standing long jump. The sample was not normally distributed (T1, *p* = 0.206; T2, *p* = 0.460) through the Explore analysis of SPSS. The level of significance was set at α = 0.05. The effect size (ES) of T1 and T2 in each parameter was calculated by Cohen’d as a practical evaluation of the quantitative results. Effect size values of 0.2, 0.5, and 0.8 for small, medium, and large effect sizes [23]. Using G*Power software (G*Power 3.1, Dusseldorf, Germany) to calculate the Statistical power of T1 and T2 in each parameter, the statistical significance level was set at Power = 0.8 [23].

## 3. Results

The standing long jump distance achieved following rope skipping training (T2) was significantly greater than that achieved without rope skipping intervention (T1) (z= −2.271, *p* = 0.023, ES = 0.97, Power = 0.94). At take-off, the velocity of the center of gravity in T2 was significantly higher than that in T1 (z = −2.830, *p* = 0.005, ES = 1.00, Power = 0.95), and the angle of the center of gravity in T2 was significantly smaller than that in T1 (z = −2.411, *p* = 0.016, ES = 0.59, Power = 0.56). There was no significant difference between kinematic parameters in T1 and T2 during the flight phase (*p* > 0.05). At landing, the velocity of the center of gravity in T2 was significantly higher than in T1 (z = −2.132, *p* = 0.033, ES = 0.75, Power = 0.77), and the hip joint angle in T2 was significantly smaller than in T1 (z = −1.992, *p* = 0.046, ES = 0.70, Power = 0.71) (Table 2).

## 4. Discussion

The study was designed to analyse the effect of an 8-week rope skipping intervention on standing long jump performance. Each participant carried out rope skipping in accordance with their own rhythm and with intensity reaching 65–75% of the maximum heart rate [16]. Rope skipping is a stretch-shortening cycle (SSC) that is commonly applied to improve jumping ability. To date, skipping has mainly formed part of studies of physiological responses and has rarely been employed in corrective kinematic analysis. Our findings showed that the intervention of rope skipping training can help students of male in physical education institute to increase their standing long jump performance, so we can make assumptions boldly that college students who are not in physical education institute will have a greater significant effect after intervening in rope skipping training, but this assumption needs to be further research to confirm. Besides, also found that standing long jump distance and the velocity of the center of gravity at take-off and landing were significantly increased following rope skipping training. Notably, the angle of the center of gravity at take-off and the hip joint angle at landing decreased. Ideally, the angle of the center of gravity at take-off should be between 19° and 27° to maximize long jump distance. Moreover, the forward tilt of the hip joint should be properly extended on landing to increase the velocity of the center of gravity and thus long jump distance.

Rope skipping exercises the whole body that requires rhythm, coordination, agility, speed, force and balance [5,6,7,8,9]. It has been observed in previous studies of jumping ability that beginners receiving SSC rope skipping training recorded shortened contact time with the ground, increased jump height, and changes in the contribution of each muscle group or joint angle control of the lower legs [10,24]. Both centrifugal and centripetal contraction abilities and high-power output over a short time can be improved by enhanced training. In particular, the muscle groups in the thigh and calf are stimulated during SSC associated with rope skipping. Other studies have demonstrated that the coordination and balance of elite young footballers can be effectively improved by 8-week rope skipping training (2 times per week and for 15 min), suggesting that the activity may be generally beneficial for improving sporting performance alongside specialist training [11]. Similarly, it was found that the explosive power, agility, and response time of female teenage volleyball players was improved after 12 weeks of rope skipping training (3 times per week and for 40 min) [12]. The study is considered a key performance index for volleyball players. Separate reports describe improvements in cardiovascular endurance (10.33%) and agility (3.17%) of adolescent boys following 7-week rope skipping training (3 times per week and for 15–50 min) [4]. In addition, teenagers’ body mass index can be increased and their physical fitness improved by 8 weeks of rope skipping [13].

Standing long jump is often used to assess lower body force and power [25,26]. The research results show that the standing long jump distance achieved following rope skipping training (T2) was significantly greater than that achieved without rope skipping intervention (T1) (z = −2.271, ES = 0.97). Standing long jump performance is affected by the velocity, angle, and height of the center of gravity [2,21,22,27,28,29]. Furthermore, at take-off, the velocity of the center of gravity in T2 was significantly higher than that in T1 (z = −2.830, ES = 1, Power = 0.95), and the angle of the center of gravity in T2 was significantly smaller than that in T1 (z =−2.411, ES = 0.59, Power = 0.56). Previous studies have observed that the distance of standing long jump depends on the performance of the body’s center of gravity. At take-off, the hip joint is tilted forward, and the extension postures are increased in the knee and ankle joints to strengthen the pushing force [21,29]. There was no significant difference between kinematic parameters in T1 and T2 during the flight phase (*p* > 0.05). Previous studies have shown that the ideal take-off angle of the center of gravity in the standing long jump lies between 19° and 27° [21]. In this study, the average angle of the center of gravity in T1 and T2 were 32.6° and 30.2° respectively, indicating a need for improvement. Optimal velocity and angle of the center of gravity are the critical factors during the flight phase [29]. In this study, at landing, the velocity of the center of gravity in T2 was significantly higher than in T1 (z = −2.132, ES = 0.75, Power = 0.77), and the hip joint angle in T2 was significantly smaller than in T1 (z = −1.992, ES = 0.70, Power = 0.71), our findings supported the principle that it is necessary to raise the velocity of the center of gravity and extend the hip joint forward to prolong the flight phase and thus increase jump distance [27,28].

At present, only male college students from physical education institute as the research participants. The research results may not be applicable to female college students from physical education institute, non-physical education college students, or people of other age groups. This is one of the limitations of this research. It is expected that more age groups will be collected in the future. Strategies, as well as non-Physical School students, were used as research participants to explore more of the value offered by rope skipping.

## 5. Conclusions

In this study, movement analysis confirmed that an 8-week rope skipping intervention enhances standing long jump performance. The velocity of the center of gravity at take-off and landing were significantly improved. However, the angle of the center of gravity at take-off was larger than the ideal angle of 19° to 27° required for maximum distance. In addition, the study confirmed the requirement for forward tilt of the hip joint at landing to increase the velocity of the center of gravity and hence long jump distance. The detailed kinematic analysis described here provided further evidence of the benefits of integrating non-specialized and specialized training activities to enhance athletic performance and offers a contribution to movement theory and practice.

## Figures and Tables

**Table 1:** An 8-Week Rope Skipping Intervention.

Item	Duration (min)	Training Content
Warm-up Exercises	10	800-m jogging and dynamic stretching
Double-Leg Jump	1	Hold the handles of the rope in each hand Jumping feet together Double-leg vertical jumps
Take a break	1	heart rate had decreased to 120 beats/minute at the rest interval
Single-Leg Jump (right foot)	1	Hold the handles of the rope in each hand Single foot vertical jumps
Take a break	1	heart rate had decreased to 120 beats/minute at the rest interval
Single-Leg Jump (left foot)	1	Hold the handles of the rope in each hand Single foot vertical jumps
Take a break	1	heart rate had decreased to 120 beats/minute at the rest interval
Side to Side and Front to Back Jumps	1	Hold the handles of the rope in each hand Jump left Jump right Jump forward Jump backward
Take a break	1	heart rate had decreased to 120 beats/minute at the rest interval
Alternating Leg Jump Rope	1	This is a single leg jump rope activity similar to jogging Hold the handles of the rope in each hand Make sure to use a light knee and ankle motion while jumping on the balls of the feet While jumping, alternate the feet every time the rope passes through
Take a break	1	heart rate had decreased to 120 beats/minute at the rest interval
Cool-down Exercises	10	Static Stretching

**Table 2:** The performance of the standing long jump and the parameters of each stage. (Mean ± SD) (N = 15).

	T1	T2	z	d	Effect Size	Power
Performance of the standing long jump						
Standing long jump distance (m)	2.34 ± 0.18	2.51 ± 0.17	−2.271 *	0.97		0.94
The instant of the take off						
Velocity of the center of gravity (m/s)	3.39 ± 0.30	3.69 ± 0.30	−2.830 **	1.00	large	0.95
Angle of the center of gravity (deg)	32.60 ± 4.60	30.20 ± 3.28	−2.411 *	0.59	medium	0.56
Height of the center of gravity (m)	86.27 ± 6.71	84.47 ± 6.37	−1.363	0.27	small	0.17
Hip joint (deg)	159.08 ± 10.90	159.01 ± 9.18	−0.245	0.01	-	0.05
Knee joint (deg)	160.78 ± 8.80	161.11 ± 8.17	−0.245	0.04	-	0.05
Ankle joint (deg)	115.94 ± 23.35	121.92 ± 18.29	−1.083	0.28	low	0.17
Flight phase						
Flight time (s)	0.45 ± 0.04	0.45 ± 0.04	−0.633	0.00	-	0.05
The instant of the landing						
Velocity of the center of gravity (m/s)	3.62 ± 0.28	3.82 ± 0.25	−2.132 *	0.75	medium	0.77
Angle of the center of gravity (deg)	−38.51 ± 5.80	−38.06 ± 3.91	−0.035	0.09	-	0.06
Height of the center of gravity (m)	57.88 ± 7.07	62.51 ± 7.11	−1.293	0.65	medium	0.65
Hip joint (deg)	64.86 ± 18.10	53.91 ± 10.60	−1.992 *	0.70	medium	0.71
Knee joint (deg)	130.78 ± 11.48	136.33 ± 9.03	−1.922	0.53	medium	0.48
Ankle joint (deg)	101.52 ± 12.39	100.78 ± 10.89	−0.524	0.06	small	0.06

** *p* < 0.01; * *p* < 0.05.
